# Large-scale single-virus genomics uncovers hidden diversity of river water viruses and diversified gene profiles

**DOI:** 10.1093/ismejo/wrae124

**Published:** 2024-07-08

**Authors:** Yohei Nishikawa, Ryota Wagatsuma, Yuko Tsukada, Lin Chia-ling, Rieka Chijiiwa, Masahito Hosokawa, Haruko Takeyama

**Affiliations:** Computational Bio Big-Data Open Innovation Laboratory (CBBD-OIL), AIST-Waseda University, 3-4-1 Okubo, Tokyo 169-0082, Japan; Research Organization for Nano & Life Innovation, Waseda University, 513 Waseda Tsurumaki-cho, Tokyo 162–0041, Japan; Computational Bio Big-Data Open Innovation Laboratory (CBBD-OIL), AIST-Waseda University, 3-4-1 Okubo, Tokyo 169-0082, Japan; Graduate School of Advanced Science and Engineering, Waseda University, 2-2 Wakamatsu-cho, Shinjuku-ku, Tokyo 162-8480, Japan; Graduate School of Advanced Science and Engineering, Waseda University, 2-2 Wakamatsu-cho, Shinjuku-ku, Tokyo 162-8480, Japan; Graduate School of Advanced Science and Engineering, Waseda University, 2-2 Wakamatsu-cho, Shinjuku-ku, Tokyo 162-8480, Japan; Research Organization for Nano & Life Innovation, Waseda University, 513 Waseda Tsurumaki-cho, Tokyo 162–0041, Japan; Computational Bio Big-Data Open Innovation Laboratory (CBBD-OIL), AIST-Waseda University, 3-4-1 Okubo, Tokyo 169-0082, Japan; Research Organization for Nano & Life Innovation, Waseda University, 513 Waseda Tsurumaki-cho, Tokyo 162–0041, Japan; Graduate School of Advanced Science and Engineering, Waseda University, 2-2 Wakamatsu-cho, Shinjuku-ku, Tokyo 162-8480, Japan; Institute for Advanced Research of Biosystem Dynamics, Waseda Research Institute for Science and Engineering, Graduate School of Advanced Science and Engineering, Waseda University, 3-4-1 Okubo, Shinjuku-ku, Tokyo 169-8555, Japan; Computational Bio Big-Data Open Innovation Laboratory (CBBD-OIL), AIST-Waseda University, 3-4-1 Okubo, Tokyo 169-0082, Japan; Research Organization for Nano & Life Innovation, Waseda University, 513 Waseda Tsurumaki-cho, Tokyo 162–0041, Japan; Graduate School of Advanced Science and Engineering, Waseda University, 2-2 Wakamatsu-cho, Shinjuku-ku, Tokyo 162-8480, Japan; Institute for Advanced Research of Biosystem Dynamics, Waseda Research Institute for Science and Engineering, Graduate School of Advanced Science and Engineering, Waseda University, 3-4-1 Okubo, Shinjuku-ku, Tokyo 169-8555, Japan

**Keywords:** single-virus genomics, environmental viruses, droplet microfluidics, whole genome amplification, DNA viruses

## Abstract

Environmental viruses (primarily bacteriophages) are widely recognized as playing an important role in ecosystem homeostasis through the infection of host cells. However, the majority of environmental viruses are still unknown as their mosaic structure and frequent mutations in their sequences hinder genome construction in current metagenomics. To enable the large-scale acquisition of environmental viral genomes, we developed a new single-viral genome sequencing platform with microfluidic-generated gel beads. Amplification of individual DNA viral genomes in mass-produced gel beads allows high-throughput genome sequencing compared to conventional single-virus genomics. The sequencing analysis of river water samples yielded 1431 diverse viral single-amplified genomes, whereas viral metagenomics recovered 100 viral metagenome-assembled genomes at the comparable sequence depth. The 99.5% of viral single-amplified genomes were determined novel at the species level, most of which could not be recovered by a metagenomic assembly. The large-scale acquisition of diverse viral genomes identified protein clusters commonly detected in different viral strains, allowing the gene transfer to be tracked. Moreover, comparative genomics within the same viral species revealed that the profiles of various methyltransferase subtypes were diverse, suggesting an enhanced escape from host bacterial internal defense mechanisms. Our use of gel bead-based single-virus genomics will contribute to exploring the nature of viruses by accelerating the accumulation of draft genomes of environmental DNA viruses.

## Introduction

Environmental viruses (primarily bacteriophages) are the most abundant and diverse biological agents [1]. Through infection of host cells, lytic viruses have a major impact on nutrient and energy cycles [2], whereas lysogenic viruses contribute to the diversification of the host genomes as one of the major mobile genetic elements [3]. Viruses are abundant in aquatic environments (10^4^–10^8^ particles/mL) [4, 5], and various studies have researched the genome sequencing of aquatic DNA viruses. Currently, the most commonly used method for viral genome sequencing is viral metagenomics, which has been applied to various environmental samples, including soil [6], sea sediment [7], and freshwater [8]. Although viral metagenomics has collected diverse viral sequences and viral databases are being expanded, the diversity of the environmental viruses and their functions remain unclear [9]. Current metagenomics has difficulty constructing viral genomes because they are highly mosaic, with frequent genomic mutations and recombination, reducing the efficiency of metagenomic assembly [10]. As genome variation can cause critical changes in viral ecology, including determining host ranges [11], the large-scale acquisition of viral genomes and elucidation of their diversity are essential for understanding their functions.

Single-virus genomics, which uses fluorescence-activated cell sorting (FACS) for viral particle isolation and whole genome amplification (WGA), is an alternative method for obtaining viral sequences, including genomic microdiversity [12]. To date, collecting viral single-amplified genomes (vSAGs) has revealed double-stranded (ds) DNA viral populations dominating oceans, which have been overlooked by metagenomics [13]. However, isolating nanometer-sized viral particles using FACS is technically challenging, and the efficiency of genome amplification was limited to 20% of sorted samples at most [13–15]. Therefore, the number of recovered vSAGs has been limited, and only the most abundant populations were recovered [16].

This study proposes a new single-virus genome sequencing platform using microfluidic-generated gel beads. Microfluidic gel beads have been used in diverse studies as reaction vessels for high-throughput single-cell analysis, including genome amplification of prokaryotic cells [17–20]. Gel beads encapsulate viral particles in a high-throughput manner, allowing genome amplification of >10^5^ viruses in a single tube at a time. Following WGA, gel beads with amplified DNA were isolated by FACS, which improves the sorting efficiency compared to the conventional FACS of viral particles. Validation using model viruses showed that high-throughput single-virus genomics provides accurate sequence information. In addition, we demonstrate the power of this platform to uncover the genomic diversity of river water viruses.

## Materials and methods

### Packaging of model viruses

The LAMBDA INN *in vitro* Packaging Kit (NIPPON GENE Co, Ltd, Toyama, Japan) was used for packaging Lambda DNA (48.5 kb) and Charomid 9-42 DNA (42 kbp) (NIPPON GENE Co, Ltd). For the *in vitro* packaging, we used 200 ng of Lambda DNA and 100 ng of Charomid DNA. After packaging, the two viral suspensions were treated with 10 units of DNase I (NIPPON GENE Co, Ltd) for 25°C, 2 h in 50 μL of the reaction mixture. The DNase reaction was terminated by adding 100 mM EDTA (Invitrogen Co, Carlsbad, CA, USA). As a control, packaging extracts without DNA packaging were used for droplet encapsulation after DNase I treatment. For calculating the concentration of each viral particle, each viral suspension was individually encapsulated into gel beads, subjected to WGA, and the fluorescence-positive rate was calculated. Then, two viral suspensions were mixed in equal volume and subjected to gel bead-based WGA and high-throughput sequencing.

### Preparation of viral suspensions from river water

We collected 3 L of surface river water from the Komatsuka Bridge (N 35.42.43, E 139.43.24) in the Kanda River basin, a typical urban river in the Tokyo metropolitan area, on 21 May 2021. The Kanda River is a managed river for flood control reservoir, and it flows directly into Tokyo Bay [21]. The river water at the sampling site is freshwater, and the river depth is <1 m. The river water was filtered under reduced pressure through a 5-μm filter (Merck Millipore, Milan, Italy) and a 0.22-μm filter (Merck Millipore) to remove large particles. The filtrate was used to concentrate viruses by suction filtration or chemical flocculation with FeCl_3_. We performed each method as previously reported with some modifications [22, 23].

In suction filtration, 200 mL of 0.22-μm filtrate was filtered under reduced pressure through an Anodisc filter (0.02-μm pore size, Merck Millipore) to trap the viral particles on the membrane. The Anodisc filter was transferred to a 50-mL tube (Iwaki Science Products Department, Iwaki Glass Co Ltd, Chiba, Japan), and 500 μL of the 0.02-μm filtrate was added. The filter was then crushed and stirred to resuspend the viral particles. After centrifugation at 3000 × g and 4°C for 3 min, 400 μL of supernatant was collected in a 1.5-mL tube (Axygen Biosciences, Hangzhou, China). In chemical flocculation, we followed the previously reported method [23]. Sterile iron chloride solution was added to 300 mL of the 0.22-μm filtrate to a final concentration of 10 mg Fe/L, left for 1 h at room temperature, and filtered through a 0.8-μm filter (Merck Millipore). The 0.8-μm filter was soaked in 500 μL of 1× oxalic acid resuspension buffer (0.1 M EDTA, 0.2 M MgCl_2_, 0.2 M Ascorbate, in 0.125 M Tris buffer, pH = 6.5) and incubated in Hulamixer agitation (222DS, LabNet, Edison, New Jersey, USA) at 4°C for 16 h to resuspend the viruses.

The viral suspensions concentrated by the two methods were subjected to chloroform treatment (1:10 (v/v)) to remove possible DNA carrier vesicles [24] and DNase I treatment using the same protocol as that for the model viruses, and then to gel bead-based WGA.

### Viral particle imaging

We sent 50 μL of the viral suspensions prepared by method (I) and method (II) to Hanaichi Ultra Structure Research Institute (Aichi, Japan). Negative staining transmission electron microscopy (TEM) images at a magnification of 100 000× were taken using a JEM-1400 microscope (JEOL Ltd., Tokyo, Japan).

### Extraction of viral metagenomic DNA

We used 200 μL of viral suspension collected in method (I) and extracted viral metagenomic DNA using a DNeasy Blood & Tissue Kit (QIAGEN, Hilden, Germany) with bead beating. For bead beating, 600 mg of zirconia beads (YZB01, Yasui Kikai, Osaka, Japan) in a 2-mL tube (Funakoshi Co, Ltd, Tokyo, Japan) were used. A cycle of 1 min on/off was repeated three times with a bead crusher (μT-12, Taitec Corp, Saitama, Japan). The concentration of the extracted DNA was calculated using a Qubit Fluorometer (Invitrogen Co. Carlsbad, CA, USA).

### Microfluidic gel bead-based single-virus genome amplification

We mixed 30 μL of viral suspensions after DNase I treatment in a 1:1 ratio with 3% low-melting-point agarose (A5030, Sigma Aldrich) in Dulbecco’s Phosphate Buffered Saline (DPBS, Thermo Fisher Scientific, Waltham, MA, USA), and gel beads with a particle size of 30 μm were generated using an On-chip Droplet Generator (On-chip Biotechnologies, Tokyo, Japan). Droplets encapsulating viruses were processed according to previously published methods (single amplified genome in gel beads: SAG-gel) [25, 26] and lysis of viral particles (1 mg/mL proteinase K [Promega, Madison, WI] and 0.5% SDS [Wako, Tokyo, Japan] in DPBS at 40°C for 2 h). DNA denaturation and WGA were performed using a REPLI-g Single Cell Kit (QIAGEN). After three times washing with DPBS, genomic amplification within the gel beads was confirmed by staining with SYBR Green I (Thermo Fisher Scientific) and fluorescence microscopy (Keyence Co, Osaka, Japan). The rate of fluorescence-positive gel beads was determined, and the viral concentrations were calculated. When the fluorescence-positive rate exceeded 20%, viral suspensions were diluted with DPBS and gel beads were generated again to prevent co-encapsulation of multiple viruses.

The SYBR-stained gel beads were suspended in DPBS and were subjected to FACS (BD FACSMelody, BD Biosciences, Franklin Lakes, NJ, USA) in 384-well plates. Based on the results of microscopic observations, the population with higher fluorescence intensity was isolated. For the mixed samples of model viruses, 240 fluorescence-positive gel beads were isolated. For river water samples, 768 fluorescence-positive gel beads were sorted for the two viral preparation methods (1536 in total).

### Sequence library preparation and high-throughput sequencing

For viral metagenomic sequencing, 100 ng of viral metagenomic DNA was used for library preparation using the TruSeq DNA PCR-free high-throughput library prep kit (QIAGEN). The library was sequenced on a NextSeq2000 System (P2 Reagents, 150 × 2 cycles; Illumina, San Diego, CA, USA).

A sequencing library was prepared using the QIAseq FX DNA library kit (QIAGEN) for single-virus genome sequencing. During library preparation, the amplified DNA in gel beads was fragmented and released from gel beads. Gel beads were also lysed during the heating process of the DNA fragmentation and library amplification steps. The library of model viruses was sequenced on a NextSeq2000 System (P1 Reagents, 150 × 2 cycles, Illumina), and the library of river water samples was sequenced on a MiSeq System (Reagent Kit v3, 300 × 2 cycles, Illumina). We used different processes for DNA extraction, library preparation, and sequencing platforms.

### Sequence analysis of model viruses

Sequence reads were trimmed using fastp with the “-q 25 -r” option. A total of 104 samples containing >50000 trimmed sequence reads were used for downstream analysis. Using Bowtie v2.3.4.1 [27] as the default parameter, the sequence reads were mapped to the integrated reference sequences of Lambda DNA (RefSeq, Accession No. NC_001416.1) and Charomid 9-42 DNA (provided by NIPPON GENE Co, Ltd). The sequence reads were also mapped to *Escherichia coli* K-12 strain reference genome (ATCC 10798) in the same manner. Mapping statistics were calculated using Samtools v1.13 to evaluate cross-contamination and genome coverage [28]. The Gini coefficient was calculated to evaluate amplification bias, which was generated for each sample using the bam-lorenz-coverage tool [29] based on the mapping results in the BAM files. Gini coefficient takes values between 0 and 1, with smaller values indicating less amplification bias [30, 31].

### Sequence analysis of river water samples

#### Construction of viral metagenome-assembled genomes and viral single-amplified genomes

To construct viral metagenome-assembled genomes (vMAGs), metagenomic sequence reads were quality-filtered to remove low-quality reads or reads derived from adapter sequences using fastp v0.20.1 [32] with the parameter “-q 25 -r”. Quality-filtered sequence reads were subjected to viral contig assembly using metaviralSPAdes [33] with default parameters. The acquired viral contigs were further filtered using VirSorter2 v2.0 [34] as the default parameters. The quality of the resulting 104 viral contigs was evaluated using CheckV v0.7.0 [35], and 100 viral contigs with >0% completeness were used as vMAGs.

For constructing vSAGs, we used different processes from vMAGs. Sequence reads were quality-filtered using fastp v0.20.1 [32] and de novo assembled by SPAdesv3.15.0 [36] with the parameters “--sc --careful --disable-rr --disable-gzip-output”. The contigs were filtered to detect viral contigs using VirSorter2 v2.0 with the parameters “--min-score 0.3 --provirus-off --prep-for-dramv”. We evaluated the viral contigs using a metagenomic binning tool to group the fragmented viral contigs within each gel bead rather than employing the longest viral contig within each gel bead. The comparison of vSAGs and the largest viral contigs is provided in the Supplementary Information and Fig. S5. First, to evaluate the relative abundance of each viral contig in the metagenomic raw reads, the quality-filtered metagenomic sequence reads were mapped to all viral contigs using bwa-mem v0.7.17 [37] with the default parameters. Then, MetaBAT2 v2.15.6 [38] was run with the parameters “--minS 40 --maxP 99 --minClsSize 10000 --maxEdges 500 --saveCls --noBinOut --unbinned” and viral contigs were binned within each gel bead based on their relative abundance and tetranucleotide frequency. For each resulting bin, genome completeness was evaluated using CheckV v0.7.0 [35]. Because CheckV does not accept multiple sequence inputs, the viral contigs within each bin were concatenated using poly-N linkers before evaluation. Finally, only the bins with the highest completeness (> 0%) were selected as vSAGs and used for subsequent analyses. To reduce the risk of viral sequence contamination, contigs in the other bins were excluded. We evaluated the number of gel beads with contigs classified into multiple distinct bins. For the 105 of 1536 gel beads that were determined to contain no viral sequences, the origins of the sequences were investigated using the Bin Annotation Tool (BAT) v5.2.3 [39].

#### Phylogenetic analysis of viral single-amplified genomes and viral metagenome-assembled genomes

The protein-coding regions of vSAGs and vMAGs were predicted using Prodigal v2.6.3 [40] with the “-p meta” option. For all predicted gene regions, vConTACT2 v0.9.20 [41] was performed to identify the protein clusters (PCs) with “--rel-mode Diamond --db ProkaryoticViralRefSeq201-Merged --pcs-mode MCL --vcs-mode ClusterONE” options. Based on the shared patterns of PCs, vSAGs and vMAGs were clustered into viral clusters (VCs). The protein-sharing network was visualized using Cytoscape v3.9.1 [42]. The representative sequence of each VC was determined as the sequence with the highest completeness. The relative abundance of each VC in the environment was estimated by mapping quality-filtered metagenomic reads to the representative sequence. In this process, CoverM v0.6.1 (https://github.com/wwood/CoverM) was used with the “genome --mapper bwa-mem -m rpb” option to calculate the reads per base value. CoverM was also used to calculate metagenomic raw reads coverage for each vSAG with the “--mapper bwa-mem -m covered_fraction --min-covered-fraction 0” option. Next, to reveal the microdiversity within each VC, metagenomic reads were aligned to the representative sequence of each VC by running blastn [43] with the option “-evalue 0.00001 -perc_identity 50”, ensuring a query coverage of over 80%. Probability density distribution plots for the alignment results were created using seaborn’s kdeplot with the “bw_method = 0.5” option.

The viral proteomic tree was created by running ViPTree Gen v1.1.2 [44] using default parameters. A proteomic tree containing whole vSAGs and vMAGs was created using ViPTreeGen with the 1Nov2021 genomes.fa fasta file downloaded using Inphared. pl in November 2021 [45]. To assess the novelty of our sequences against existing databases, vSAGs and vMAGs were referenced to the IMG/VR v3 database (2020-10-12_5.1) [46] using blastn v2.5.0+ with “-evalue 1e-3 -culling_limit 1 -max_target_seqs 1” according to Minimum Information about an Uncultivated Virus Genome (MiUViG) criteria (ANI ≥ 95%, AF ≥ 85%) [47].

Phylogenetic prediction of vSAGs and vMAGs was performed using two methods described in a previous study [46]. Firstly, predicted proteins were compared to all viral proteins in RefSeq (obtained on 22 March 2022) using DIAMOND v2.0.14 [48] with the option “blastp --evalue 1e-5 --query-cover 50 --subject-cover 50 --max-target-seqs 10000”. For sequences with >30% protein hits on the viral RefSeq protein, consensus affiliation was determined using a voting system based on the best hit of the protein with a 50% majority rule. Secondly, taxonomic classification was determined based on the detection of 588 marker genes in the VOG database v97 [46]. The predicted protein and 588 VOG hmm profiles were compared using hmmscan v3.3.2 [49] with the “-E 1e-2” option, with a minimum score of 40 and a maximum *E* value of 1e-05. When conflicting marker genes were detected, the annotation was limited to the level at which the lineages matched.

#### Identification of auxiliary metabolic genes

Functional predictions of vSAGs and vMAGs were made using DRAM-v v1.2.4 [50] with default parameters. We identified 643 genes as auxiliary metabolic genes (AMGs) with an auxiliary score of 1–3 in the amg_summary.tsv file output by the distillation process of DRAM-v. For AMGs without gene description, gene description annotations were added with the priority of KEGG, CAZY, or Pfam ID hits. The possession patterns of AMGs were compared for medium- or high-quality vSAGs and vMAGs classified into VCs.

The amino acid sequences of bacterial transglutaminase-like cysteine proteinase (BTLCP) and terminase large subunit (TerL) were aligned using mafft v7.475 [51] with “—auto –reorder” parameters. Ambiguously aligned positions were trimmed using TrimAL v1.4. rev22 [52] with “-automated1” parameter. Phylogenic trees of these genes were reconstructed using iqtree v2.1.2 [53] with the option “-bb 1000 –alrt 1000” from the trimmed file. The maximum-likelihood trees were reconstructed by automatic model selection, and the topology was supported by ultrafast bootstrap analysis with 1000 replicates and SH-like aLRT analysis with 1000 replicates. After midpoint rooting using gotree [54], the phylogenetic trees were visualized using iTOL v6 [55].

#### Analysis of the viral operational taxonomic unit: species-level clustering and detection of gene insertion in vOTU572

Viral operational taxonomic units (vOTUs) were identified by running aniclust with default settings according to the MiUViG species criteria (ANI ≥ 95%, AF ≥ 85%) [35, 47]. In the analysis of seven sequences classified as vOTU572, the genes were clustered into Orthologous Groups (OGs) using Orthofinder v2.5.4 [56] with default parameters. The OGs shared by all vSAGs were assigned as core genes, and the other OGs were designated as flexible genes. Comparative genomics were performed using gggenomes v0.9.5.9 (https://github.com/thackl/gggenomes) and Clinker v0.0.23 [57]. Sequence read mapping and multiple alignments were performed using bwa-mem v0.7.17 as default parameters [37].

## Results

### Gel bead-based single-virus genomics enables high-throughput and accurate viral genome sequencing

**Figure 1:**
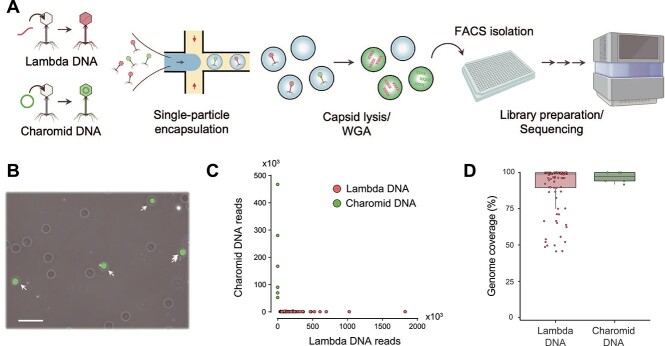
Gel beads enable high-throughput WGA of viruses at the single-particle level. (A) Workflow of single-virus genomics. Viruses are encapsulated into 30-μm of microfluidic droplets with ultra-low melting temperature agarose. After the solidification of agarose, each virus captured in a gel bead proceeded to undergo capsid lysis and WGA. Gel beads with amplified DNA are isolated with FACS and proceeded to the library preparation and high-throughput sequencing. (B) Microscopic image of gel beads after WGA. The amplified viral DNA is stained with SYBR Green I, with a scale bar of 100 μm. (C) Comparison of the number of sequence reads mapped to the Lambda and Charomid reference sequences. Samples were labeled with the reference with the most sequence reads to be mapped. Only 4 of the 96 gel beads contained >1% of the other viral sequence reads, suggesting a low risk of DNA contamination. (D) Genome coverage by the sequence reads derived from each gel bead.

Viruses were encapsulated into 30 μm-diameter microfluidic gel beads at the single-particle level, followed by capsid lysis and WGA. This platform applies modified protocols of the previously reported bacterial single-cell genome sequencing method (single amplified genomes in gel beads: SAG-gel [25, 26]) for viruses. As proof of principle, viruses packaging two different types of DNA (Lambda and Charomid 9-42) were prepared. Gel bead-based WGA was performed using a mixture of the two viruses (Fig. 1A). After 3 h of WGA, DNA amplification was confirmed in gel beads (Fig. 1B). The average fluorescence-positive rate was 3.2%. The viral concentration was calculated to be 9.4 × 10^7^ pfu/μg (*n* = 3), which was slightly lower than the kit reference value (1 × 10^8^ pfu/μg for standard Lambda DNA). In contrast, when we encapsulated the packaging extract without DNA packaging as a control, the fluorescence-positive rate was <0.1% (*n* = 3). High-throughput sequencing of amplified DNA in gel beads yielded a median of 186 K trimmed sequence reads for 104 quality-filtered samples, 92.3% (96/104) of which predominantly contained sequence reads mapped to the reference. In the remaining 7.7% (8/104) of samples, 89.4 ± 2.3% of sequence reads were mapped to the *E. coli* genome. Because the sequence results of control samples without DNA packaging also showed a predominance of *E. coli* reads, we consider these sequences to be derived from the packaging extract. All sequenced gel beads had 95.5 ± 5.9% of their quality-filtered sequence reads mapped preferentially to either Lambda DNA or Charomid DNA, with only four gel beads containing >1% of the other viral sequence reads (Fig. 1C and Table S1). This result suggests that the risk of contamination of the other viral sequences could be reduced by encapsulating dispersed viral particles at appropriate concentrations. The genome coverage was 84.8 ± 24.6% (*n* = 90) for Lambda DNA and 96.6 ± 3.8% (*n* = 6) for Charomid DNA (Fig. 1D). A preliminary estimation of the two viral concentrations showed the number of viral particles containing Lambda DNA was 8.2–12 times larger than that of Charomid DNA. The sequencing results showed a 15 times higher number of gel beads containing Lambda DNA than Charomid DNA. This result indicates that our platform collects each type of viral sequence roughly according to the actual composition of viral particles. The average Gini coefficient was 0.38 for Lambda DNA and 0.088 for Charomid DNA, showing less values compared to other bacterial single-cell genome amplification methods [58]. The lower Gini coefficient in Charomid DNA may be due to the characteristic genome structure of it [59].

### Large-scale single genome sequencing of viruses from river water

**Figure 2:**
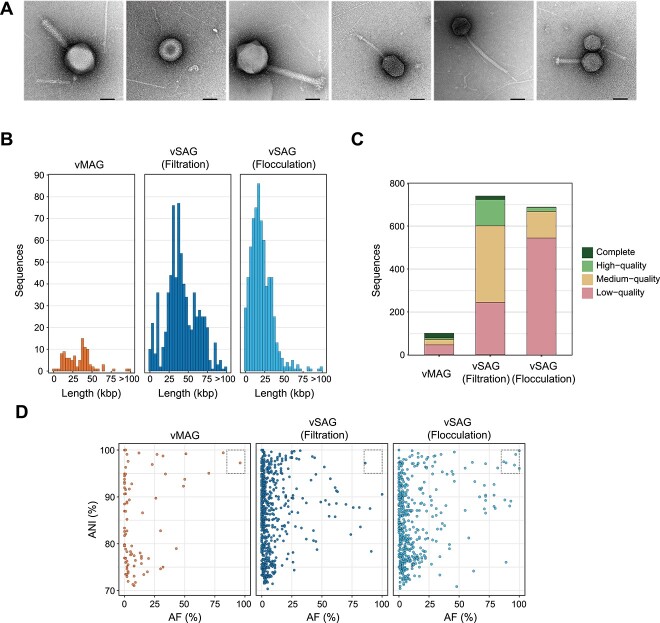
Sequence quality evaluation of vSAGs and vMAGs collected from river water. (A) Negative staining TEM images of viral suspensions were taken at a magnification of 100 000×. Various shapes of virus-like particles were recovered from river water. Most particles are dispersed, whereas some seem to be physically attached. The scale bar is 500 nm. Viruses were collected using two different methods ((I) suction filtration and (II) flocculation with FeCl_3_), and single-virus genomics was performed with each viral suspension. Metagenomic DNA was extracted from the viral suspensions collected by method (I). After high-throughput sequencing and the construction of vSAGs and vMAGs, (B) the sequence length, (C) the collected number and the estimated quality by CheckV, and (D) the sequence similarity against reference sequences in the IMG/VR v3 database were evaluated. If the average nucleotide identity (ANI) was >95% and the alignment fraction (AF) was >85%, the viral sequences were judged to be known sequences (dashed square).

We collected surface river water from an urban river in Tokyo and prepared viral fractions using two methods: (I) suction filtration [22] and (II) flocculation with FeCl_3_ [23]. These two methods are commonly used for virus particle recovery, and their recovery efficiencies were examined. TEM images of the viral fractions showed various morphologies of virus-like particles, including those with tail structures (Fig. 2A).

Following WGA, the fluorescence-positive rate of gel beads was ≤13.2% and the calculated concentration of DNA virus in the river water was 9.2 × 10^5^ particles/mL by method (I) and 2.6 × 10^5^ particles/mL by method (II). A total of 1536 DNA-amplified gel beads (768 for each method) were isolated and proceeded to high-throughput sequencing, yielding 12.4 Gb sequence reads by method (I) and 11.6 Gb by method (II), respectively. Although genome sequencing of environmental viruses poses a risk of contamination, such as physical aggregation of viral particles, there are currently no analytical tools to assess contamination in viral sequences. Therefore, we employed the metagenomic binning tool to construct vSAGs to exclude potentially contaminating sequences. If contigs derived from a single gel bead were classified into multiple distinct bins, contigs in the bin with the highest completeness were used to construct the vSAG, and contigs in the other bins were excluded. We found 54.2% (775/1431) of the gel beads had contigs classified into multiple distinct bins. Excluded contigs do not directly indicate viral sequence contamination because contigs derived from one viral species could be classified into multiple bins due to the lack of sequence information. Finally, we recovered 1431 vSAGs from 740 gel beads (96.3% of the total) by method (I) and 691 gel beads (90.0% of the total) by method (II), demonstrating a significantly higher recovery rate than conventional single-virus genomics [13–15]. A total of 57.2% (819/1431) of vSAGs comprised the single largest contig; the rest comprised multiple contigs grouped by binning process. Of the 105 gel beads with no viral sequences detected, 54 had small data sizes (<5000 paired reads), and most of the remaining sequences were determined to contain fragmented reads of bacteria or viruses. In the metagenomic sequencing that uses extracted DNA from the viral suspension in method (I), we recovered 16.8 Gb reads and 100 vMAGs.

The number of vSAGs or vMAGs per 1 Gb of sequence reads for method (I), method (II), and for metagenomics was 59.7, 59.6, and 5.95, respectively. The median length of vSAGs was 39 781 bp for method (I) and 18 296 bp for method (II), and that of vMAGs was 34 985 bp (Fig. 2B and Table S2). In the quality assessment by CheckV [35], 496 (67.0%) of vSAGs by method (I), 144 (20.8%) of vSAGs by method (II), and 54 (54.0%) of vMAGs were medium- or high-quality (Fig. 2C and Table S2). No viral sequence was detected in 6.8% (105/1536) of gel beads.

As a result of referring to IMG/VR v3 database, 99.9% (739/740) and 99.1% (685/691) of vSAGs with method (I) and method (II), respectively, were determined novel at the species level, with only seven sequences corresponding to the reference (Fig. 2D). In the vMAGs, 99.0% (99/100) were determined novel. Of the two virus concentration methods, method (I) exhibited a longer median length and higher genome quality than method (II), consistent with a previous study [23].

### Single-virus genomics recovers highly diverse and low-abundance viral sequences

**Figure 3:**
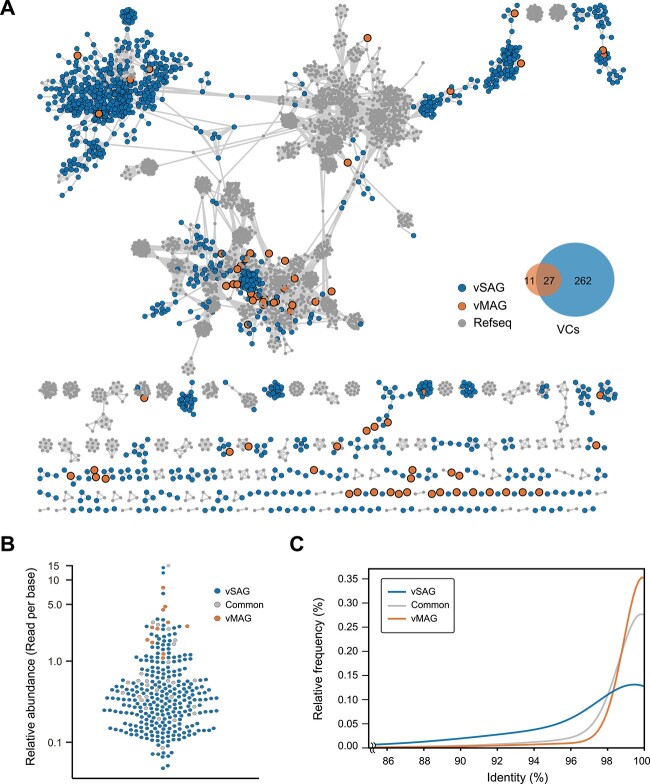
Protein-sharing network of 1431 vSAGs and 100 vMAGs. (A) Protein-sharing network analysis of 1431 vSAGs and 100 vMAGs with RefSeq reference database using vConTACT2. Only the sequences sharing at least one PC with other sequences are represented. (B) Relative abundance of vSAGs and vMAGs in the metagenomic raw reads. The vertical axis is the number of mapped metagenomic sequence reads to the representative sequences, expressed as the logarithm of reads per base. (C) Metagenomic sequence reads recruitment patterns (also referred to as diversity curves) for the representative sequences of each VC. Curves represent the percentage of recruited sequence reads at each nucleotide identity value [13].

Phylogenetic diversity of vSAGs and vMAGs was evaluated by constructing VCs based on a protein-sharing network (Figs 3A and S1), consistent with the genus-subfamily level classification. In total, we detected 289 VCs from 873 vSAGs (223 VCs from method (I) and 153 VCs from method (II)) and 38 VCs from 50 vMAGs (Table S2). There were 27 VCs detected in common between vSAGs and vMAGs. The number of VCs clustered with the reference sequence was only 14 in vSAGs and seven in vMAGs, suggesting that numerous novel sequences at the genus-subfamily level were obtained.

Taxonomic classification was conducted using the methodology employed in the IMG/VR v3 database, which references the 2019 ICTV Release. Of the 50 vMAGs that clustered into VCs, 20% (10/50) were taxonomically assigned at the family level, all of which were tailed dsDNA viruses (*Caudoviricetes*), including *Siphoviridae*, *Myoviridae*, and *Podoviridae* (Fig. S2A and Table S2). In contrast, only 6.5% (57/873) of vSAGs clustered into VCs were taxonomically assigned. Most sequences were assigned to dsDNA viruses (Fig. S2B), whereas two vSAGs were assigned as *Mimiviridae* and four as *Lavidaviridae,* which are giant viruses and virophages, respectively. In addition, 10 single-stranded (ss) DNA viruses, including *Circoviridae* and *Microviridae*, were recovered because ssDNA was also amplified by WGA [60]. Of the 10 737 genes hit by the RefSeq viral protein, 10 324 (96.2%) were best-matched to bacteriophage genes, suggesting that most of the viral sequences were from bacteriophages.

We then assessed the relative abundance of vSAGs and vMAGs in the metagenomic raw reads. vSAGs contained viral sequences of diverse abundance, including relatively low abundance viral sequences (Fig. 3B). Species-specific recruitment patterns for each VC [13] indicated that vSAGs recovered viral populations with both microdiversity and macrodiversity whereas vMAGs had a relatively low sequence diversity (Fig. 3C). Because the metagenomic raw reads contained 68.9% (986/1431) of the vSAG sequences with >50% coverage, we suggest that most of the viral sequences were missed during the process of metagenomic assembly.

### Identification of auxiliary metabolic genes shared among viruses of different lineages

We detected viral AMGs from vSAGs and vMAGs that were of medium- or high-quality and clustered into VCs to assess how viral infection modulates host metabolism. We detected 280 AMGs of 70 types in 161 vSAGs and 16 AMGs of 10 types in 11 vMAGs (Table S3). The highest number of AMGs per vSAG was 10, and the most abundant functional category of AMGs was affiliated with organic nitrogen metabolism (Fig. S3A, B).

**Figure 4:**
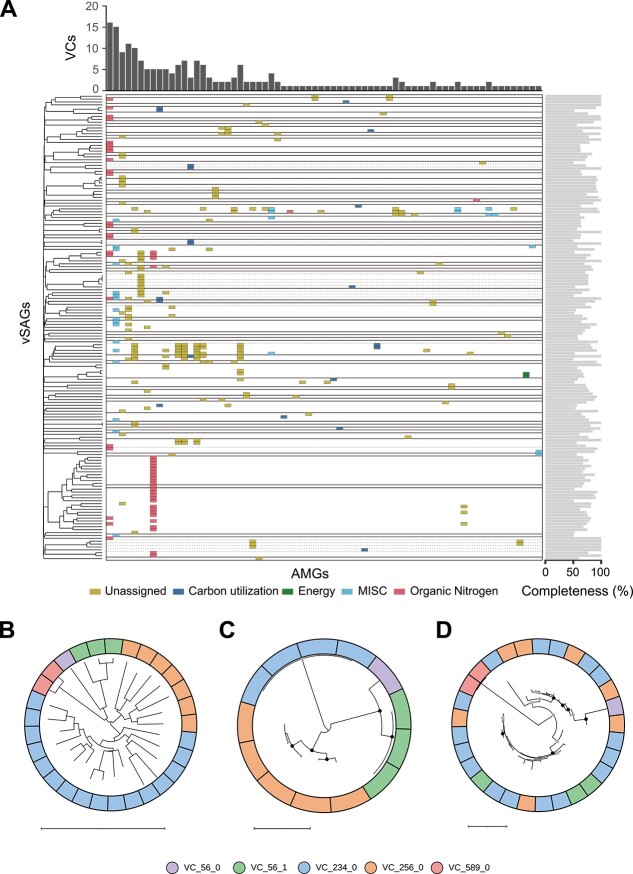
Identification of AMGs commonly detected from multiple VCs. (A) The distribution profiles of AMGs among vSAGs. Each row represents a single vSAG, and each column represents a single AMG. Straight lines separate each VC, and subclusters within a VC are separated by dashed lines. The AMG categories were determined by DRAM-v. The left side of the figure shows the phylogenetic tree of vSAGs, and the right-side bar plot shows the completeness of each vSAG. The histogram at the top shows the number of VCs containing each AMG, showing 33 types of AMGs from multiple VCs and 37 types of AMGs from a single VC. (B) Proteomic tree created by ViPTree of vSAGs with BTLCP detected. Maximum-likelihood phylogenies of (C) TerL and (D) BTLCP. Branches with bootstrap support by SH-like aLRT higher than 80% and ultrafast bootstrap 95% are indicated by black dots. If those branches were only supported by SH-like aLRT higher than 80%, they are shown in gray. Each color of the outer circle represents a different VC value. Each scale bar means tree scale: 1.

The PC profiles were then evaluated to determine gene transfer among viruses. We detected 9754 PCs from 494 vSAGs and 34 vMAGs with medium- or high-quality. Although a previous report using metagenomic contigs showed that distinct VCs share few protein groups [61], large-scale single-virus genomics revealed that 30.7% (2993/9754) of PCs were shared in multiple distinct VCs (Table S4). There were 233 AMGs of 33 types from multiple VCs, which corresponds to 2.3% (68/2993) of PCs (Fig. 4A). In contrast, 47 AMGs of 37 types were detected from only a single VC, which corresponds to 0.84% (57/6761) of PCs. These results suggest that AMG-containing PCs were more frequently distributed among VCs (*P* = 0.01, Fisher’s exact test). Next, a phylogenetic analysis of vSAGs harboring BTLCP, the most frequently identified AMG, was conducted. The proteomic tree created by ViPTree showed clades corresponding to each VC (Fig. 4B). In addition, the phylogenetic tree of TerL, which is often used in phylogenetic analysis, showed clades corresponding to each VC with robust bootstrap support (Fig. 4C). In contrast, the phylogenetic tree of BTLCP genes did not cluster by VCs but branched within diverse clades (Fig. 4D). As some clades were supported by sufficient bootstrap values (SH-like aLRT ≥80%), BTLCP may have been acquired via horizontal gene transfer after the divergence of these VCs.

### Comparative genomics reveals the profiles of methyltransferase (MTase) are diversified within the same viral species

**Figure 5:**
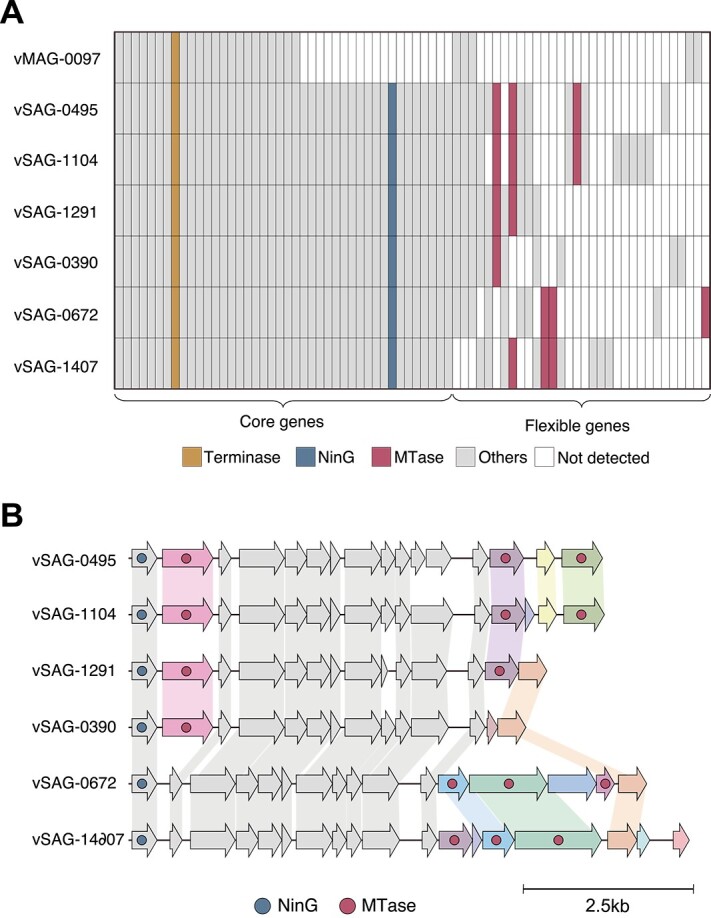
Comparative genomics within the same vOTU (vOTU572). (A) An OGs matrix where each row represents a single OG and columns represent six vSAGs and one vMAG grouped into vOTU572. The top sequence is derived from the vMAG, and the others are derived from vSAGs. The presence or absence of a gene is indicated by color. Gray columns indicate genes that were not annotated, and white columns indicate the genes that were not detected. (B) Alignment of six vSAGs partial genomes. Open reading frames are represented by block arrows. Gray-colored arrows indicate genes in the core region, and other colored arrows indicate genes in the flexible region. Six different MTase subtypes were detected in vOTU572. The lead depth in the flexible region of vSAGs was 258.8 on average and 23.3 at the lowest.

Clustering was performed at the vOTU level, which corresponds to the viral species level, to assess the viral genomic microdiversity. Based on the MIUViG criteria [47], 1197 vOTUs were generated. Of these, 11.5% (138/1197) of vOTUs consisted of multiple viral sequences. We focused on vOTU572, obtained in one from vMAG with a completeness of 51.0% and six from vSAGs with an average completeness of 93.7%. vOTU572 is novel at the genus level compared with the IMG/VR v3 database [46] and forms an independent VC. The seven sequences within vOTU572 were very similar, as the sequence identity of the TerL was 100%, and ANI was >98.9%. In contrast, the profile of OGs revealed the presence of two genomic regions: highly conserved regions (core) and regions with structural diversity (flexible) (Fig. 5A). Most of the regions recovered by the vMAG belonged to the core regions, and the flexible regions were lost. Although metagenomic raw reads were also mapped to flexible regions (Fig. S4), the most flexible region was not detected in metagenomic contigs. In addition, even when only metagenomic reads corresponding to the flexible regions were extracted and re-assembled, the flexible region was not constructed. These results suggest that the reads corresponding to the flexible region were sequenced but missing during assembly. Genes were annotated in 11.9% (5/42) of the core regions and 22.6% (7/31) of the flexible regions. The core region included genes with TerL, bacteriophage lambda NinG protein (PF05766), and putative peptidoglycan binding domain (PF01471), whereas we identified multiple methyltransferase (MTase) subtypes in the flexible regions. The sequence alignment of each vSAG revealed that various MTase subtypes were inserted into the genomes (Fig. 5B). As the viral MTase contributes to escape from the bacterial restriction-modification systems by methylating their DNA [62], diversifying the pattern of MTase by frequent homologous recombination suggests a strain-level adaptation strategy of viruses against the internal immune mechanisms of host bacteria.

## Discussion

Environmental viruses are more diverse than environmental bacteria, and genomic mutations occur frequently, leaving many challenges for high-resolution genome analysis [9]. Single-virus genome sequencing using FACS has been performed; however, it has some difficulties in sorting accuracy, and previous reports have recovered only a few dozen viral sequences mainly because of the low success rate of WGA [13–15]. Here, we developed a gel bead-based single-virus genomics platform and recovered a total of 1431 viral sequences from 93.2% of the isolated gel beads. Our platform dramatically improved the efficiency and throughput of viral genome sequencing and recovered a large number of diverse viral sequences, including those with low relative abundance in the metagenomic raw reads. In contrast, the results of mapping metagenomic raw reads to vSAGs imply that metagenomic assembly is hampered in vMAG construction by viral genomic microdiversity, which is recognized as “the great metagenomics anomaly” [10, 54]. As 91.8% of the viral sequences obtained in this study were novel at the family level, accumulating vSAGs could help in the phylogenetic classification of environmental viruses.

High-throughput genome sequencing enabled high-resolution detection of PCs commonly detected in different VCs, allowing the tracing of gene distribution among viruses. As a higher percentage of AMGs were commonly detected in multiple distinct VCs compared to other PCs, these AMGs are suggested to play a significant role in virus survival. For example, sequence homology analysis suggested that BTLCP can be exchanged via co-infection with the same bacterial host and transferred among distinct VCs. As BTLCP is suggested to be involved in catalyzing post-translational protein modification [55], modifying prokaryotic surface structures [55], and biofilm formation [63], the acquisition of such AMGs could be advantageous for host bacterial adaptation to the environment.

Large-scale single-virus genomics has enabled obtaining closely related but diverse viral sequences. Comparative genomics revealed frequent homologous recombination of various MTase subtypes within the same vOTU. A recent study reported that viral host specificity is not determined by proteins involved in viral adhesion to the cell membrane but rather by the internal defense mechanisms of the host bacteria [64]. Furthermore, a study analyzing nearly identical viral genomes from distant aquatic ecosystems also reported frequent transfer of MTase and suggested that MTase protects viruses against host-encoded restriction-modification systems [65]. Our results imply that viruses with multiple MTase subtypes could enhance their ability to escape bacterial internal defense mechanisms, broadening their host ranges. It has been reported that genes involved in viral immunity are frequently horizontally transferred among bacteria [66]. Correspondingly, this study confirmed that genes suggested to be involved in host adaptation are widely distributed among viruses.

We developed a gel bead-based single-virus genomics platform to reveal gene transfer among diverse viruses and microdiversity within the same vOTU in river water viruses. As our platform can recover various single-virus genome sequences, large-scale comparative genomics has enabled the detection of genetic recombination, which is the most significant factor in virus diversification [65, 67]. In contrast, 39% (558/1431) of the acquired vSAGs did not cluster into a VC, suggesting that larger genome sequencing would be required for a comprehensive protein-sharing network analysis. Although the current platform allows obtaining >1400 viral sequences in a single sequence run, further development, such as multiplexed barcoding [68], will be necessary. Also, WGA using phi29 may experience amplification bias for genomes with varying sizes/linearity/GC content. In addition, careful handling is required to reduce the risk of viral sequence contamination. Potential causes of viral genomic contamination are the physical adhesion of viral particles and the co-encapsulation of multiple viruses, so it is necessary to observe the virus particles in suspension and keep the ratio of DNA-amplified gel beads under a certain level. Further improvements are also needed in the method of viral particle concentration. Moreover, bioinformatics tools should be developed to evaluate viral sequence contamination. Future accumulation of viral sequences may allow single-copy marker genes to be defined within specific lineages, which could be used as an indicator of viral contamination.

We performed a snapshot analysis of the viral community; however, time-lapse sampling would allow the analysis of the temporal variation of the viral community and horizontal gene transfer occurring at that moment. Although viral genome sequencing with long-read sequencers has been demonstrated, it requires a substantial amount of water to extract DNA of sufficient quality [69, 70]. In contrast, our platform can perform genome sequencing from a few hundred milliliters of water, making it easier to perform analyses on temporal variation in viral communities. Furthermore, cross-referencing single-cell genomes of environmental bacteria with vSAGs will enhance our understanding of virus-host interactions, including host range and bacterial immunity against viruses. Our single-virus genomics platform will contribute to elucidating the unknown functions of environmental viruses.

## Supplementary Material

Revise_ISME_SI_v5_wrae124

Supplementary_Table1_wrae124

Supplementary_Table2_wrae124

Supplementary_Table3_wrae124

Supplementary_Table4_wrae124

## Data Availability

All raw sequencing data described in this article are available on NCBI SRA under BioProject accession number PRJNA863333. Within this BioProject, the sequences of 1431 vSAGs and 100 vMAGs have been deposited at GenBank under the accession KIDV00000000. If more information is needed, please contact the corresponding author.
