# Supplementary material for: Large-scale single-virus genomics uncovers hidden diversity of river water viruses and diversified gene profiles
Source: ISME J. 2024 Jul 8;18(1):wrae124. doi: 10.1093/ismejo/wrae124 (PMC11283719; doi:10.1093/ismejo/wrae124)
Supplement: Revise_ISME_SI_v5_wrae124 [file revise_isme_si_v5_wrae124.docx]

**Supplementary Information**

**The comparison of vSAG and the largest viral contig constructed from each gel bead**

For constructing vSAGs, we employed the metagenomic binning tool to group the fragmented viral contigs within each gel bead to reduce the risk of viral sequence contamination rather than employing the largest viral contig within each gel bead. To evaluate the effect of binning viral contigs, we compared vSAG with the largest viral contig obtained from each gel bead in terms of sequence length and completeness estimated by CheckV. In method (Ⅰ), the median length of the vSAGs was 39.8 kbp, while that of the largest contigs was 33.6 kbp. In method (Ⅱ), the median length of the vSAGs was 18.3 kbp, while that of the largest contigs was 14.4 kbp (Fig. S5A). The increase in the sequence length also increased the completeness evaluated by CheckV. In method (Ⅰ), the number of sequences judged as medium- or high-quality was 362 (48.9%) when using the largest viral contig, and the number of vSAGs judged as medium- or high-quality was 496 (67%). In method (Ⅱ), the number of sequences judged as medium- or high-quality was 91 (13.2%) when using the largest viral contig, and the number of vSAGs judged as medium- or high-quality was 144 (20.8%) (Fig. S5B). In the metagenomic sequencing using extracted DNA from the viral suspension in method (Ⅰ), the average length of vMAGs was 35 kbp, and 54% of vMAGs were judged as medium- or high-quality. These results suggest that vMAG showed slightly superior to the largest viral contig within each gel bead when comparing the average sequence quality. On the other hand, because single-virus genomics has higher efficiency in viral sequence recovery, the absolute number of medium- or high-quality viral sequences was higher in the largest viral contigs than in metagenomics.

The number of viral contigs detected from each gel bead was evaluated before and after the binning process. By applying a binning tool, the average number of viral contigs in each gel bead decreased from 7.0 to 2.0 in method (Ⅰ) and from 4.4 to 1.7 in method (Ⅱ) (Fig. S5C). The above results suggest that the binning process effectively groups the viral contigs within each gel bead.
